# EduNet: A New Video Dataset for Understanding Human Activity in the Classroom Environment

**DOI:** 10.3390/s21175699

**Published:** 2021-08-24

**Authors:** Vijeta Sharma, Manjari Gupta, Ajai Kumar, Deepti Mishra

**Affiliations:** 1 Centre for Development of Advanced Computing (C-DAC), Pune 411008, India; vijeta.it@gmail.com (V.S.); ajai@cdac.in (A.K.); 2 DST Center for Interdisciplinary Mathematical Sciences, Institute of Science, Banaras Hindu University, Varanasi 221005, India; 3 Department of Computer Science (IDI), NTNU—Norwegian University of Science and Technology, 2815 Gjøvik, Norway

**Keywords:** artificial intelligence, classroom activity recognition, classroom monitoring, EduNet dataset, education

## Abstract

Human action recognition in videos has become a popular research area in artificial intelligence (AI) technology. In the past few years, this research has accelerated in areas such as sports, daily activities, kitchen activities, etc., due to developments in the benchmarks proposed for human action recognition datasets in these areas. However, there is little research in the benchmarking datasets for human activity recognition in educational environments. Therefore, we developed a dataset of teacher and student activities to expand the research in the education domain. This paper proposes a new dataset, called EduNet, for a novel approach towards developing human action recognition datasets in classroom environments. EduNet has 20 action classes, containing around 7851 manually annotated clips extracted from YouTube videos, and recorded in an actual classroom environment. Each action category has a minimum of 200 clips, and the total duration is approximately 12 h. To the best of our knowledge, EduNet is the first dataset specially prepared for classroom monitoring for both teacher and student activities. It is also a challenging dataset of actions as it has many clips (and due to the unconstrained nature of the clips). We compared the performance of the EduNet dataset with benchmark video datasets UCF101 and HMDB51 on a standard I3D-ResNet-50 model, which resulted in 72.3% accuracy. The development of a new benchmark dataset for the education domain will benefit future research concerning classroom monitoring systems. The EduNet dataset is a collection of classroom activities from 1 to 12 standard schools.

## 1. Introduction

AI topics include everything from video monitoring and surveillance to human activity recognition and behavioral analysis. There is increasing demand for the development of domain-specific datasets that perform deep research in specific fields. ImageNet, for example, is a largescale database that allows collecting and annotating a large number of image datasets [1], as it has thousands of image categories. In contrast, human action datasets in videos are comparatively less.

An emerging trend in AI research is detection and recognition in online videos or real-time camera feed. Nowadays, in schools, classrooms are equipped with CCTV cameras, indicating the possibility of automatically analyzing classroom activity. Therefore, the development of a new benchmark dataset for the education domain is needed; it will benefit related research concerning classroom monitoring systems. The lack of a dataset designed explicitly for student–teacher classroom activities is a significant obstacle in this research area. Video data generated from CCTV cameras installed in classrooms can utilize AI technology to automatically recognize human activity in live classroom environments.

One focus of the proposed dataset concerns the granularity levels of human activities. Every day, large amounts of video data are uploaded to online media platforms, and data captured from mobile and surveillance cameras accelerate the rate of video data generation. In a recent survey (https://www.statista.com/statistics/259477/hours-of-video-uploaded-to-youtube-every-minute/, accessed on 19 August 2021), YouTube reported that over 500 h of video were uploaded to their servers every minute. It was observed that, despite the rapid growth in video data generation, computer vision research is lagging in regard to automatically recognizing human activities. Due to the lack of domain-specific video datasets, the research in this area has not advanced much. The majority of existing action recognition datasets consist of everyday activities of humans. Some specific human action recognition datasets, such as Sports-1M [2] and UCF Sports [3], are specially designed for sports activities. There is no action recognition dataset specifically designed for classroom monitoring, which plays a vital role in boosting the education system.

Therefore, we developed a new dataset—EduNet—with 20 “actions” in the classroom environment, exclusively containing student–teacher scenes. We collected related videos from YouTube and actual classrooms from various backgrounds, such as classrooms from rural areas, urban areas, age groups, and class standards. The constructed dataset consists of 7851 video clips, with a minimum of 200 clips in each action category. Each video clip ranges from 3 to 12 s. The total duration of the video is approximately 12 h. The dataset consists of real videos from YouTube, uploaded by users worldwide, and includes multiple scene angles, cluttered backgrounds, and recordings from actual classrooms.

We analyzed the particular characteristics of the classroom scenes and the technical difficulties in recognizing actions. Valid annotations were conducted for the researchers to have a clear understanding. Due to the particularity of classrooms, our datasets enhance the credibility of new algorithms. Moreover, we provide a comparison of characteristics within the current benchmark action recognition datasets. Additionally, we present a thorough performance analysis of EduNet using the I3D-ResNet-50 action recognition model. The results show that our dataset is viable and reliable.

The availability of large, labeled image datasets has enabled continued breakthroughs in machine learning research. However, video datasets for particular domains need large-scale datasets for deep research, such as AlexNet [4], VGG16 [5], GoogLeNet [6], ResNet [7], etc. Classroom teaching has a core role in education. Obtaining student–teacher activity is key toward measuring the quality of education. With the help of massive amounts of classroom teaching videos generated in the era of big data, student and teacher classroom activities can be automatically recognized by AI technology to evaluate and improve the quality of classroom teaching. We observed that the required dataset had not been developed in the classroom scene to date. Thus, little progress has been made in AI classroom activity. Therefore, the development of a classroom activity monitoring dataset was required. The EduNet dataset is purely research-centric. Data were collected by considering the different perspectives and algorithmic challenges. The focus was on school classroom activity monitoring. Thus, the actions belonged to 1 to 12 standard students and their respective teachers. This complex ground-level dataset indicates the need for higher algorithmic research requirements.

Presently, the majority of human action recognition video datasets focus on certain types of activities, i.e., cooking [8,9], sports [2,3], or simple actions [10]. These datasets are bound with limitations, such as a small number of human activity categories (daily routine activity, indoor activities, outdoor activities, etc.), a small number of samples (short length clips, i.e., less than 3 s), and limited domains (common activities, sports, kitchen, etc.). A recent survey [11] listed 26 open action recognition video datasets in the following four categories—(i) action level datasets, (ii) behavior level datasets, (iii) interaction level datasets, (iv) group activities level datasets. However, this rapid development also demands different levels of advancement in video datasets.

Few datasets, specially developed for human action recognition (HAR) [12,13,14], show the incremental development of action classes, from 400 to 600 and 700, which prove the continuous enhancement of HAR video datasets.

### 1.1. Problem Statement

There is a high demand for live camera feeds to recognize human actions, e.g., for surveillance purposes. However, there is a niche for action recognition in the classroom environment. The lack of such a dataset is problematic. Therefore, we propose the EduNet dataset, which consists of 7851 well-annotated video clips of student–teacher actions in classroom environments.

### 1.2. Motivation

Education is a fundamental right, and the quality education is equally important. In the era of AI, research into the education system can boost the quality of education by utilizing action recognition from video data. For example, a student dataset was prepared [15] to recognize, detect, and caption student behaviors in a classroom; it contained 11 action categories. Another work (for student action recognition) was conducted by the authors of [16], which included seven action categories. Finally, to identify the teacher activity, the authors of [17] prepared an instructor activity video dataset, called IAVID-1.

Moreover, the authors of [18] proposed a teacher action dataset, TAD-08, from a large amount of videos featuring teacher behavior recognition. Our primary goal of EduNet dataset started with motivation. Due to the absence of a “classroom activities” type of dataset, we enthusiastically built a dataset for classroom activities, to recognize teacher and student activities and to monitor active participation in the classroom (which plays a vital role in quality education).

### 1.3. Contribution

EduNet, a classroom activity monitoring dataset, contains 20 unique action classes. Each class has a minimum of 200 clips extracted from YouTube videos and recorded from a Nikon DSLR D5600 camera (Nikon Corporation, Tokyo, Japan) in a real classroom environment of 1 to 12 standard schools. The total number of video clips was 7851, approximately 12 h long. EduNet is the largest (and perhaps most realistically available) dataset in the classroom environment to date, to the best of our knowledge, containing both student and teacher actions. Each clip is manually annotated and validated to ensure consistency. We conducted an experiment on the popular human action recognition model—Inflated 3D Convolutional Neural Network (I3D) [14]. We compared the performance of EduNet with two popular datasets—UCF101 and HMDB51—while implementing the I3D-ResNet-50 model.

### 1.4. Research Questions

Table 1 presents the research questions (RQs) that we address in this work.

The rest of the paper is organized as follows. Section 2 provides a brief background. Section 3 explains the related work in this area. Section 4 provides an overview of the EduNet dataset. Section 5 elaborates the experimental details of EduNet on the benchmark action recognition model. Section 6 discusses the results. Finally, Section 7 concludes our research work with future directions.

## 2. Background

The impact of AI in the education field is already gaining popularity, with the creation of several advanced applications. For example, one application could involve intelligent classroom monitoring, by analyzing the activities of students and teachers in the classroom environment, which is different from regular or daily routine activities. The majority of (popular) human action recognition datasets are available for individuals to research/analyze everyday activities. A specially designed dataset perfects the application. Therefore, we developed the EduNet dataset, used by the computer-vision algorithm, which, in the future, could efficiently use classroom activity monitoring (similar to applications).

### 2.1. Human Action Recognition (HAR)

The primary objective of human activity recognition is to accurately describe human actions and their interactions from a previously unseen data sequence. It is often challenging to accurately recognize human activities from video data due to several problems, e.g., dynamic background and low-quality videos from cameras installed at a long-distance range, lighting, multi-subject interaction, and occlusion. Many applications, such as video surveillance, human–computer interaction, human behavior characterization, robotics, etc., require multiple activity recognition methods. Here actions may or may not be performed throughout the entire duration of the video. Technically, an action can be defined as a spatiotemporal sequence of human body movements, and videos are a collection of a set of images arranged a specific order. These image sequences are referred to as frames. Thus, the fundamental goal of action recognition is to process the input video clips to recognize the subsequent human action frames. Ideally, video data are a combination of spatial and temporal aspects. A spatial part are the “frames” extracted from the video clips and “temporal” involves the sequence of these frames at the time series. Real-time HAR application requires differentiating ambiguous actions, such as standing vs. bending, running vs. jogging, etc. These are complicated tasks and might need more than one frame’s information to identify them accurately.

### 2.2. Benchmark HAR Datasets

In simple words, benchmarking is one way to discover the best performance being achieved. In regard to benchmark video datasets for action recognition—these datasets are adequately prepared, annotated, validated, and proven to be accurate, when compared to contemporary datasets, to identify human actions. Many action recognition algorithms were top-ranked and achieved higher accuracy on these well-prepared datasets. That is why these datasets are termed benchmark datasets. A minimum of 26 human action video datasets [11] are available to date, but HMDB51 [19] and UCF101 [20] are the most popular benchmark datasets among computer-vision researchers. Recently, Kinetics datasets [12,13] have also become popular choices for researcher (for HAR), especially when utilizing the harness for pre-training of the model (with these datasets) for better accuracy.

### 2.3. HAR Deep Learning Models

In the past few years, deep learning techniques [4,5,6,7] have accelerated in the area of image data, particularly with the advent of the ImageNet dataset. Various deep learning techniques have been developed for video data, particularly human activity recognition in benchmark datasets. Unlike handcrafted feature-based approaches [21,22], deep learning-based models can simultaneously learn visual features, feature representations, and classifiers. Deep learning architectures have different variants, but the most attractive model for vision-based HAR is CNN and RNN, which have achieved very promising results on benchmark video datasets. In an experiment [2], action recognition was performed from videos using stacked video frames, such as input to the network. One different, very novel approach was introduced by [23], a two-stream CNN. One stream is dedicated to the spatial feature of the video, whereas the second stream focuses on the temporal feature. A 3D convolution network [24] performed well when compared to a 2D two-stream network. A novel approach [14] proposed a new two-stream inflated 3D ConvNet (I3D) for action recognition in the video by inflating a 2D two-stream network into 3D [25]. Apart from CNN, neural network architectures, such as recurrent neural networks (RNNs), long short-term memory (LSTM) [26], have outperformed on video data for human action recognition. Reference [27] used multilayer long short-term memory (LSTM) networks to learn high-level features of video sequences for action recognition. Reference [28] proposed a lattice-LSTM, which extends LSTM by learning independent hidden state transitions of memory cells for individual spatial locations. This method effectively and efficiently increases the model dynamic ability across time for human action recognition. Reference [29] proposed activity recognition of solitary elders via sensors data and two-layer framework consisting of coarse-grained and fine-grained deep learning methods. In another approach [30], a gait-appearance-based methodology demonstrated successful results in pedestrian re-identification.

In our experiment, we chose the I3D model [14] to evaluate the performance of the EduNet dataset.

## 3. Related Work

With rapid advancement in AI application development, building datasets are also considered immediate needs. AI algorithms heavily depend on standard and “up-to-the-mark” datasets; a successful model relies on benchmark datasets. Unfortunately, existing action recognition video datasets are cumbersome, with limitations such as lack of domain-specific dataset, fewer video clips under each class, and limited action categories.

This section presents some standard human action recognition video datasets, which are popular in the computer vision research community and similar to the EduNet dataset. For example, the Hollywood dataset [31] is a collection of videos clipped from Hollywood movies. Professional actors perform 12 action categories, having more realistic and natural scenes than contemporary video datasets [19,20]. UCF sports [2] and Olympic sports [32] are explicitly developed for sports-related action recognition tasks. These types of video datasets are considered the baseline for developing intelligent applications in the sports domain. They increase the action complexity and challenging recognition tasks due to highly articulated sporting activities, complex backgrounds, and camera angles. HMDB51 [19] datasets, compiled from movies, YouTube videos, public databases, and Google videos, have 51 action categories. Activities are divided into five categories: general facial, facial with object manipulation, general body movement, body movements for human interaction, and body movements with object interaction. HMDB51 was the most prominent action dataset at that time in 2011. A year later, in 2012, the second-largest dataset, UCF101 [20], was introduced by Central Florida, USA. This action recognition dataset was prepared to reflect real-world conditions, to monitor and analyze daily activities more closely. UCF101 categories has 101 classes of activities, divided into five groups: human–object interaction, playing musical instruments, human–human Interaction, body motion only, and sports.

Another popular sports video dataset publicly available is the sports-1M dataset [2], with about 500 sports-related categories annotated by an automatic tagging algorithm. These datasets are structured using a somewhat domain-specific activity taxonomy, as they primarily focus on sports actions, which encourage intelligent sports surveillance applications.

Charades [31] is a video dataset of daily indoor activities collected through Amazon Mechanical Turk. Videos are recorded as acting out the sentences, e.g., similar to a game of charades. The activity recognition domain is unique as it contains long action sequences. It is one of the most extensive public datasets with continuous action videos. Because of its variety of activities and long-duration clips, it is a challenging dataset.

To understand the actions and events in videos, the Moments in Time (MIT) dataset [33] was released with more than “800,000” videos. It is also a challenging dataset with state-of-the-art, accurately annotated action clips. Another dataset, Something–Something [34], is an extensive collection of fine-labeled video clips of humans performing daily routine activities with household objects in a controlled environment. The dataset also serves to collect common activities of humans, it does not explicitly focus on a specific activities. A different concept was employed when developing Epic Kitchen-55 [9], annotating first-person (egocentric). In this dataset, each action is labeled as a combination of a verb and a noun, e.g., “cut vegetable,” “wash utensil,” etc., still, it is heavily imbalanced. The extended version of this dataset contains 100 classes, later released, named Epic-Kitchen-100. AVA [35] is a dataset for spatiotemporal localization of atomic visual actions. This dataset consists of 80 different unique visual actions, which shows a baseline for the development of intelligent applications to identify kitchen-related activities.

The Kinetics-400 dataset [13] focuses on human actions (rather than activities or events), making it different from its contemporary datasets. Kinetics is two times larger than previous benchmark datasets, HMDB-51 and UCF-101, having more than 400 clips for each class. The primary feature of Kinetics is its categorization, such as (i) person actions (singular)—punching, drinking, drawing, laughing, etc.; (ii) person–person actions—kissing, hugging, shaking hands, etc.; and (iii) person–object actions—mowing the lawn, opening gifts, mopping, washing dishes, etc. An extension of the Kinetics human action dataset from 400 classes to 600 classes was further released as Kinetics-600 [12]. A year later in 2019, a dataset with 700 action classes was released as Kinetics-700 [14]. The main purpose for the Kinetics dataset was to become the ImageNet equivalent of video data. Currently, Kinetics has become the baseline dataset for benchmarking human action models. A similar dataset from EduNet is the student class behavior dataset [15]. The student class behavior dataset analyzes student behavior during master’s courses. Li et al. [36] developed a dataset to analyze student activity in the classroom with only 15 action categories. Both of these datasets do not have teacher-centric classroom activities.

EduNet has a unique objective in recognizing teacher–student activities, to improve the quality of education, from the primary level to senior secondary, targeting rural and remote areas where quality education is just a term, and not at the ground level. EduNet provides the collection of classroom activities to analyze the active participation of both teachers and students in the actual classroom environment. Student–teacher actions in the classroom are considered essential events that translate into quality education.

## 4. Dataset Details

The development of any visual recognition dataset is challenging. It takes a long time to extract clips, annotate, validate, and set a benchmark dataset for computer vision algorithms. Any custom dataset [37] preparation requires a certain number of steps to be followed. To prepare the EduNet dataset, we carefully identified the basic classroom activities performed by teachers and students. We followed the steps below in the development of the EduNet dataset.

### 4.1. Data Collection

We prepared a textual list of 20 action categories with the primary objective to search the videos of listed actions on YouTube in the first phase. Additionally, we used WordNet [38] to find the hyponyms, hypernyms, and synonyms of our classes, expanding the chances of retrieving related videos. Finally, we download the best quality user-uploaded videos in colored format. From YouTube, we successfully retrieved over 200 videos ranging from 5 to 20 min long, and we extracted approximately 3000 video clips.

In the second phase, we recorded videos against each action category in the actual classroom environment. Since this research work is non-funded, we visited multiple primary and secondary schools in rural India (north) to record the classroom videos with consent from school officials. We extracted approximately 3000 video clips from the recorded videos. The camera used was Nikon DSRL 5600. All videos were sampled to a fixed HD resolution of 1280 × 720 pixels with a frame rate of 30 fps. Dataset classes are organized into two groups: teacher centric, student centric.

### 4.2. Data Annotation

**Figure 1:**
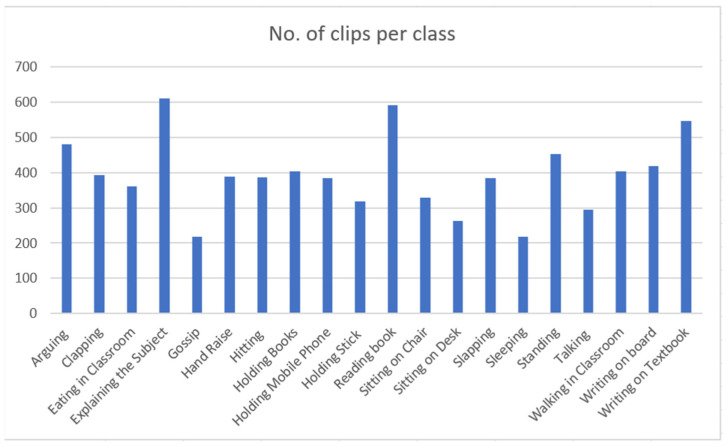
Number of clips per action class of EduNet dataset.

We verified all videos retrieved from both sources and removed those not related to the activity of our interest. With the certainty that every video must have contained at least one label, all videos were played and clipped at fixed temporal length. For the action recognition algorithm, it needed to have data with exact name to the labels. However, it is not easy to find YouTube videos only containing scenes related to our activity class. For example, when searching for YouTube videos related to the query “hand raise,” results also included videos that contained students writing on textbooks. Therefore, we decided to manually cut the video clips from entire videos within those temporal boundaries where the activity was actually performed. To ensure quality, each clip was again validated by another skilled individual. Thus, we confirmed that each clip was associated with the ground truth activity label. Figure 1 shows the number of clips in each class. The annotation file was contained in the main folder as an Annotation.txt file.

- Teacher centric labels:
  Explaining_the_subject, Hitting, Holding_Book, Holding_Mobile_Phone, Holding_Sticks, Sitting_on_Chair, Slapping, Walking_in_Classroom, Writing_On_Board.
- Student centric labels:
  Arguing, Clapping, Eating_in_Cassroom, Gossip, HandRaise, Reading_Book, Sitting_on_Desk, Sleeping, Standing, Talking, Writting_on_Textbook.

#### 4.2.1. Naming Convention

The EduNet dataset contained 20 folders, each containing the clips of one action class. The name of each clip had the following format: X_Y.mp4.

Where X is the folder name and Y is the clip number. All of the clips were in .mp4 format. For instance, HandRaise_005.mp4 corresponded to clip 5 of the action class hand raise. X is in the same order of capital and small letter in which the name of the action class is. The purpose behind this specific and easy naming convention is for the easiness of computer-vision researchers to fetch the class name by splitting the clip name. Thus, ‘_’ will split into two strings. The first string will be the action class name, and the second is the clip number.

#### 4.2.2. Dataset Split: Train, Test, Validation

The train–test split procedure is used to estimate machine-learning algorithm performances to make predictions on data not used to train the model. Additionally, a validation dataset is a sample of datum held back from training the model to estimate the model’s skills, while tuning the model’s hyperparameters and evaluate the skills of the final-tuned model, when comparing or selecting between final models. Following the current principles of dataset division, we divided the samples of each class randomly into a training set, validation set, and test set, at a ratio of 80%, 10%, 10%, as shown in Table 2.

Table 3 presents a summary of the characteristics of EduNet, in terms of the number of action classes, the total number of video clips, the minimum and maximum length of the clip, total duration of whole video clips, the minimum and the maximum number of video clips in the action class, frame rate, resolution, information of the presence of audio, and color.

**Figure 2:**
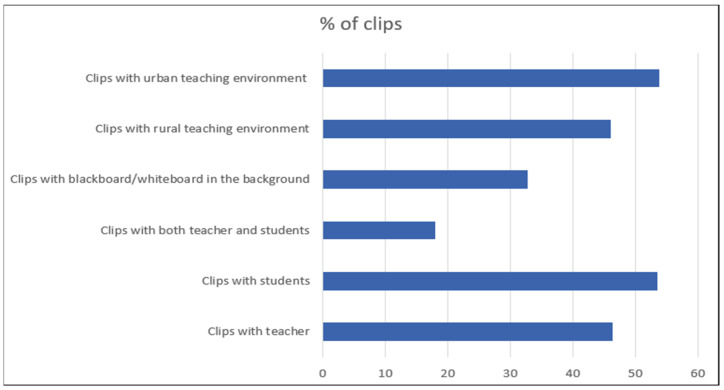
Statistics of various conditions of clips in EduNet dataset.

**Figure 3:**
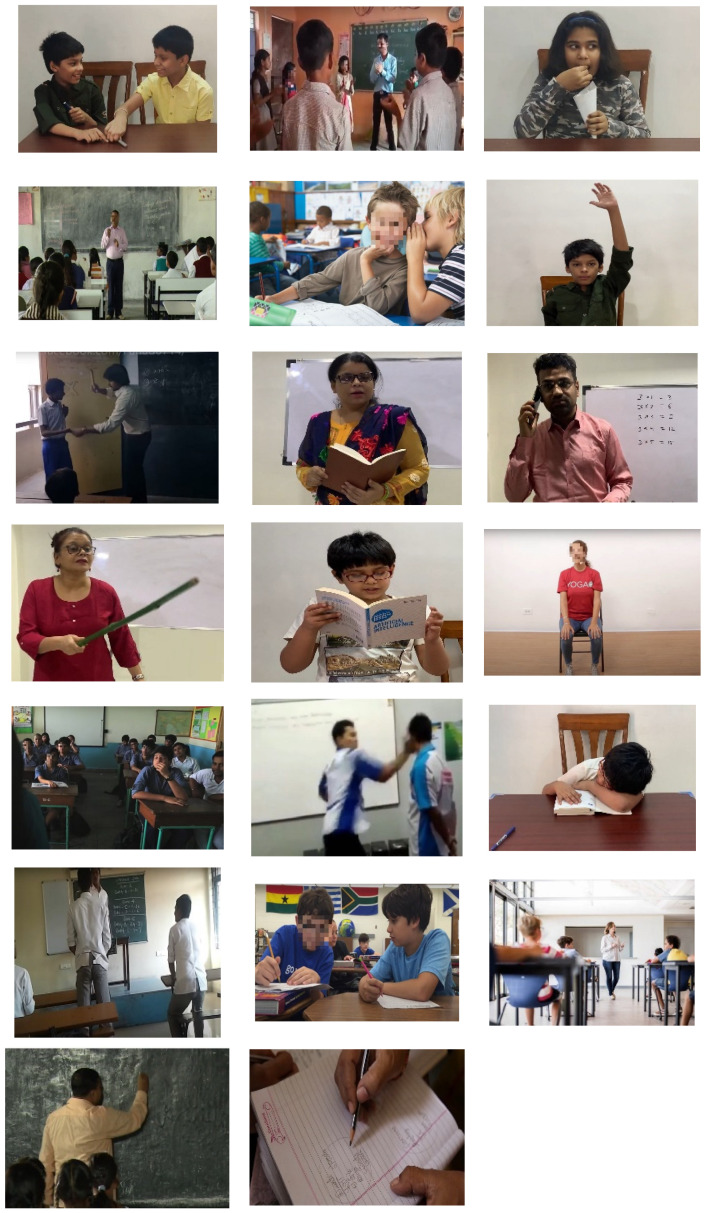
A glimpse of frames from the EduNet dataset (from top to bottom, actions are Arguing, Clapping, Eating_in_Classroom, Explaining_the_Subject, Gossip, HandRaise, Hitting, Holding_Book, Holding_Mobile_Phone, Holding_Sticks, Reading_Book, Sitting_on_Chair, Sitting_on_Desk, Slapping, Sleeping, Standing, Talking, Walking_in_Classroom, Writing_On_Board, Writting_on_Textbook).

Figure 2 shows the statistics of the EduNet dataset from various perspectives, such as the number of clips in which only the teacher scene is present, the number of clips only the student scene is present, clips in which both scenes are visible, the number of clips in which the blackboard/whiteboard is present in the background, and clips recorded from an urban/rural classroom environment. All statistics are shown in percentage values. Figure 3 presents the frames of the 20 action categories. Some of the faces in some images are blurred in order to protect their identity.

#### 4.2.3. Comparison with Other Datasets

To design the EduNet as a standard dataset, we compared its primary characteristics with several action recognition video datasets in the specific domain. Table 4 presents the domain-wise comparison of the currently available video action datasets.

From Table 4, it is observed that, to date, only two datasets involving classroom actions, refs. [15,36], are available. The dataset proposed by [36] has fewer video clips with only 817 clips. Both datasets are only student-centric. The EduNet dataset is the third proposed dataset on classroom actions; it focuses on student and teacher actions, with 7851 video clips involving 20 action classes.

#### 4.2.4. Scope

Videos were downloaded by YouTube and recorded with a DSLR camera in the live classroom. All clips were cut using Adobe Premiere Pro into varying lengths, from a minimum of 3.5 s to a maximum of 12.7 s. Most of the clips are close to 10 s long and annotated manually. All clips have a fixed frame rate and resolution of 30 FPS and 1280 × 780, respectively. The videos were saved as .mp4 files. The audio was preserved for all 20 actions at 44.100 kHz. All clips are in color format.

Multiple scopes of research on these datasets are listed below:

1. Recognition of scene-based activities of teachers and students: for example, “Explaining_the_subject” must have a background containing a blackboard. Then this activity must be detected as a teacher activity.
2. Identify activity of teachers and students distinctly: Writing_on_notebook activity is related to student activity, whereas writing_on_board is associated with the teacher activity.
3. Differentiating one activity from others: HandRaise activity performed by students must be distinguished from the Clapping activity, as the movement recognizes both by hands.
4. Categorizing positive and negative actions of the classroom: Example -Holding_Mobile_Phone shows the negative action performed by the teacher, whereas Holding_Book shows the positive action. Table 5 shows the complete list of positive and negative action categories of teachers and students, which are referred in the EduNet dataset.

## 5. Experimental Details

To avoid vast computational complexity, it is always good to utilize transfer learning. Reference [41] used transfer learning for CNN and SVM for medical image classification. It has been proven that fine-tuning is a beneficial way to obtain good models for custom datasets.

Many researchers have successfully utilized the ImageNet pre-trained model for the image classification task. A fine-tuning experiment was conducted for this paper, for classroom action classification to follow the same principle. Fine-tuning also seems to be a good practice when you do not have the computing resources to train a model from scratch for your dataset. Since there is no similar dataset currently available, we used fine-tuning to validate our dataset. We prepared Annotation.txt to pass EduNet labels and data (video clips) for the pre-trained model.

### 5.1. Machine Setup

We provided baseline results on the EduNet dataset on NVIDIA GTX 1650 Ti single GPU for training and model building. For inferencing, we used NVIDIA GTX GeForce GPU and i7 CPU, 16 GB RAM machine. We initialized the models with I3D weights pre-trained on the Kinetics-400 dataset and trained our models with a stochastic gradient descent optimizer for 40 epochs and 100 iterations per epoch. We employed a batch size of eight on a single GPU.

### 5.2. Model Architecture

We experimented using a popular I3D [14] state-of-the-art model of action recognition, which is widely accepted as a standard action recognition method for benchmarking. To optimize the model efficiently, a loss function is highly required. We used softmax cross-entropy as the loss function for the action classification task, and the momentum was set to 0.9. The initial learning rate was 0.001, and the learning rate was divided by 10 at the 20th and 40th epochs. The Inflated 3D (I3D) network is a popular 3D video classification architecture in which a 3D convolution network is employed to learn spatiotemporal information directly from video data. I3D is intentionally designed to improve the existing C3D [24,42,43] model by inflating from 2D models. Thus, we can redesign any 2D model architecture, such as Inception Net, ResNet, etc., and bootstrap the model weights from 2D pre-trained models. By following this method, we observed that this method is feasible, takes less effort, and shows better results for training 3D networks for video classification. We used the I3D-ResNet-50 model pre-trained on the Kinetics-400 dataset.

Pre-trained model i3d_resnet50_v1_custom available on MXNet (https://cv.gluon.ai/, accessed on 13 July 2021) was used for the experiment in this paper. We only excluded the top layer and replaced it with 20 classes instead of 400 output classes. Then, the network was trained as per the dataset split distribution (Table 2) in train, test, and validation, with minimal hyperparameters tuning. We limited the number of frames per clip to 250 of input data. Moreover, we skipped one frame to reduce duplication.

## 6. Results and Discussion

An EduNet dataset is a novel approach towards developing applications, such as intelligent classroom monitoring systems. We explored all of the possible ways for data collection and well annotation to clearly understand the research community. We also implemented the two-stream I3D-ResNet-50 action recognition model on EduNet to show its viability. Based on our experiment, we can now answer the RQs.

### 6.1. Analysis of Result

**Figure 4:**
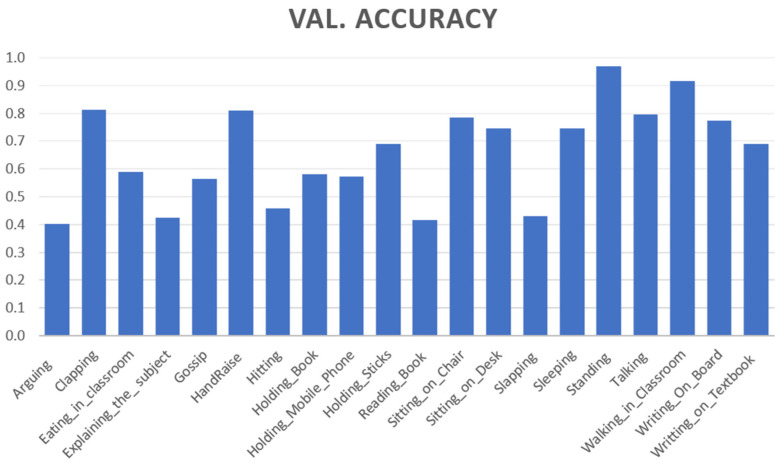
Validation accuracy of each class.

Before going into detail about the results, we will first present the analyses of the RQs, as follows:

RQ 1: we identified 20 possible teacher- and student-centric classroom actions (as shown in Figure 1) for our first RQ.
RQ 2: we obtained the conventional dataset building methodology steps: data collection, annotation with the standard naming convention, validation, and feature comparison with the existing action recognition dataset. The characteristics of the newly created EduNet dataset are summarized in Table 3.
RQ 3: we evaluated the EduNet dataset for each class performance by fine-tuning the popular two-stream I3D action recognition model. The validation accuracy of each class is shown in Figure 4 Moreover, overall accuracy was achieved at only 72.3% on the two-stream I3D model, 65.24% when applied only on the RGB video frames, and 68.8% on the optical flow data derived for the EduNet dataset. The results show that the existing algorithm does not fit well on our new domain dataset (Table 6).

**Figure 5:**
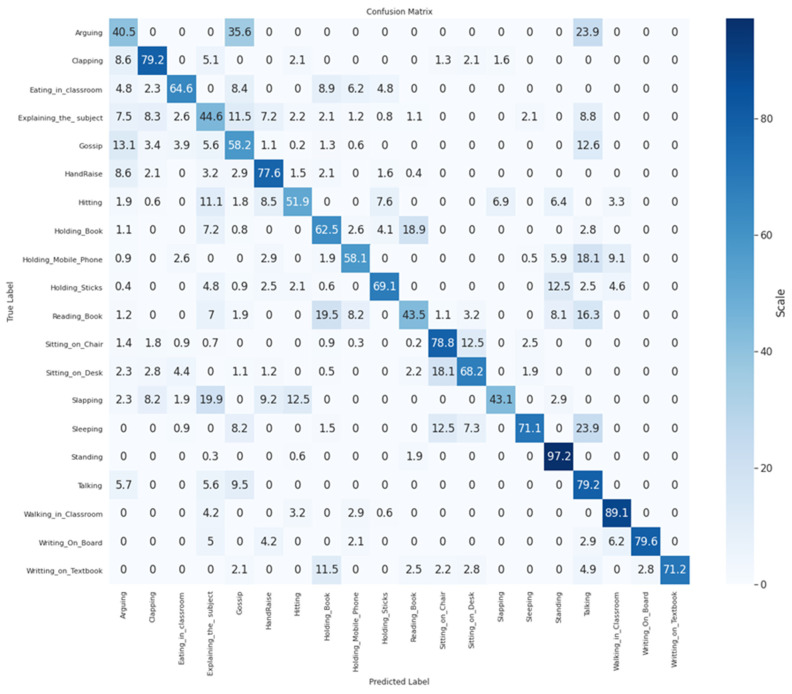
Confusion matrix.

We observed that classes—Sitting_on_Chair, Walking_in_Classroom, Writing_on_ Board, had good accuracy, because these actions are similar to the actions present in the Kinetics-400 dataset. Still, significantly newer classes, such as HandRaise, Arguing, Gossip, Hitting, had the lowest accuracy. Figure 4 presents the validation accuracy of each class, and Table 6 shows the outcome of I3D on the EduNet dataset compared to the benchmark datasets UDF101 and HMDB51. The confusion matrix in Figure 5 shows the ambiguity among Arguing, Gossip, and Talking action classes, which achieves the lower validation accuracy. These three actions are similar, until there is differentiation from the voice in the video clips. Similarly, “Holding_Mobile_Phone” and “Talking” actions have ambiguity since “Holding_Mobile_Phone” is identical to “Talking” until an object “mobile” is not detected in the video clip. Other action classes are also matched with one or more identical classes. Therefore, the I3D model could not classify it accurately.

We should also note that the two-stream I3D model we used was pre-trained on the Kinetics-400 dataset, which is a regular action dataset. I3D models pre-trained on other action recognition datasets are unavailable in the MXNet framework, which we used. Therefore, our results only show on the limited architecture. There are very few pre-trained models available for video data processing, especially action recognition, unlike image processing.

### 6.2. Threats to Validity

This section discusses the possible threats that might have affected our experiment and how we alleviated them. Validity determines whether the results from an experiment meet the requirements listed in the research method.

#### 6.2.1. Threats to Internal Validity

Internal validity refers specifically to whether an experimental condition makes a difference or not, and whether there is sufficient evidence to support the claim. Here, the main threat to internal validity is the I3D model-ResNet-50 that we used, which might not be suitable for our dataset. Therefore, it does not show good accuracy. The result may be better if we use other variants of I3D available on MXNet, such as I3D-Inception V1, pre-trained on Kinetics-400 and ImageNet, I3D-RestNet-101, pre-trained on Kinetics-400, SlowFast 4 × 16-scratch, I3D-slow-ResNet-101 pre-trained on Kinetics-700, etc.

#### 6.2.2. Threats to External Validity

External validity refers to the generalizability of the experimental outcomes. We used the transfer learning I3D-ResNet-50 network by extracting the top layer and fine-tuned it with 20 classes. The availability of computational power was a big hurdle for the experiment, i.e., the model could not be trained from scratch to learn the class labels of the self-dataset in a better way. Results of the experiment may not be exposed in a generalized way from classroom action recognition to regular actions.

#### 6.2.3. Construct Validity

Construct validity measures whether the operational definition of an experimental variable actually reflects the true theoretical meaning of the concept, initialized at the start of the study. Our experiment used the popular action recognition I3D model on the newly created EduNet dataset to check the viability. Initially, we set the 20 action classes with 7851 video clips by splitting among train, test, and validation. During each portion, we used the stipulated data to achieve maximum accuracy. However, we only obtained a 72.3% performance accuracy when the model was pre-trained with the Kinetics-400 dataset. Here, we utilized a total number of clips during our experiment as fixed at the start of the study.

#### 6.2.4. Conclusion Validity

Conclusion validity is the degree to which the conclusions we reach are credible and believable. We proposed the action recognition dataset to check the accuracy of the 20 action classes while applying the existing I3D model. As we also showed a need for the development of a new action recognition dataset in the classroom environment, we successfully created it. We tested the cruciality and difficulty level of the EduNet dataset. We observed that it only achieves 72.3% accuracy while applying the popular I3D model, which already showed high accuracy on other action recognition datasets—UCF101 and HMDB51. To show the conclusion relationship of the newly created dataset and complexity, we observed that distinguishable action categories, e.g., Arguing, Explaining_the_Subject, Hitting, Reading_Book, Slapping, are challenging to classify, even with the successful model.

## 7. Conclusions and Future Research

We developed the EduNet dataset—a challenging action recognition dataset for the classroom environment. We extracted 7851 video clips from two different sources—downloaded relevant videos from YouTube and recorded student and teacher actions from an actual classroom using a DSLR camera. Our data collection mainly focused on the school classrooms, from primary to senior secondary.

First, we identified the 20 most appropriate actions, with the clear objective to monitor and measure the quality of education in the classroom. Then, we added categories—a total of 20 student-centric and teacher-centric actions. Afterward, we manually annotated each clip with the accurate class action label. Finally, we evaluated the EduNet dataset using a standard and popular action recognition model—I3D ResNet-50 pre-trained on the Kinetics-400 dataset. Our new dataset’s baseline action recognition results achieved an overall accuracy of 72.3% when compared with other popular action recognition datasets—UCF 101 and HMDB51. The results show the development of advanced action recognition models specifically trained on classroom action datasets, which could provide better accuracy in the future.

We also compared the domain-wise existing action recognition dataset characteristics with EduNet. We found that the EduNet dataset, with 20 action classes, and over 7851 clips, make it a significantly larger dataset prepared in the classroom activity domain. This is the first dataset that mainly focuses on the action recognition of both teachers and students, which could improve the quality of education.

We should note that the task of differentiating gossip, talking, and arguing could not be done effectively without focusing on audio. Therefore, our future work will focus on developing a novel deep learning algorithm using natural language processing (NLP) to differentiate these actions accurately. The development of a novel algorithm also focuses on identifying student and teacher scenes, to recognize actions, such as “Holding_Book” and “Reading_Book” efficiently.

Because EduNet comprises of unconstrained videos downloaded from YouTube and recorded in an actual classroom environment, obstacles include poor lighting, cluttered backgrounds, and severe camera motion. Moreover, due to the change in teacher-centric and student-centric scenes, differentiating the actions of teachers and students, this task creates complexity because of the duality of work (in regard to action recognition) under the scene recognition. For computer vision researchers, the EduNet dataset will provide an opportunity to develop newer algorithms in the future, for intelligent action recognition in the classroom environment. It is assumed that this dataset will contribute, as a baseline, in the development of computer, vision-based education domain applications.

## Figures and Tables

**Table 1:** List of research questions.

ID	Research Question	Motivation
RQ1	What are the possible actions of teachers and students in a classroom environment?	Investigate the possible actions of students and teachers in the classroom to measure the quality of education.
RQ2	How can a robust and standard classroom action recognition video dataset be built?	To extract the most relevant video clips from the source videos and label them accurately against the action.
RQ3	How does one measure the viability and performance of the newly created classroom action recognition video dataset?	Choose the best-suited existing deep learning model for action recognition from videos and compare the model’s performance on the EduNet dataset with other popular datasets.

**Table 2:** Train, test, and validation split.

Class	Train	Test	Validation
Arguing	384	48	48
Clapping	315	39	39
Eating in Classroom	289	36	36
Explaining the Subject	489	61	61
Gossip	174	22	22
HandRaise	311	39	39
Hitting	309	39	39
Holding Books	323	40	40
Holding Mobile Phone	308	39	39
Holding Stick	255	32	32
Reading book	475	59	59
Sitting on Chair	263	33	33
Sitting on Desk	211	26	26
Slapping	307	39	39
Sleeping	175	30	30
Standing	363	45	45
Talking	235	30	30
Walking in Classroom	323	40	40
Writing on board	335	42	42
Writing on Textbook	438	55	55

**Table 3:** Summary of the characteristic of the EduNet dataset.

Characteristic	Description
Actions	20
Clips	7851
Min clip length	3.25 s
Max clip length	12.7 s
Total duration	12 h
Minimum clip per class	200
Maximum clip per class	593
Frame rate	30
Resolution	1280 × 720
Audio	Yes
Colored	Yes

**Table 4:** Comparisons of domain-wise action recognition datasets.

Dataset	Domain	No. of Classes	No. of Video Clips	Ref.
UCF 101	Regular action	101	13,320	[20]
HMDB 51	Facial action and body movement	51	6849	[19]
JHMDB	Regular action	21	928	[39]
Student Class Behavior Dataset	Classroom action	11	16,457	[15]
Student Action Recognition Dataset	Classroom action	15	817	[36]
Kinetics-400	Regular action	400	300,000	[13]
Kinetics-600	Regular action	600	500,000	[12]
Kinetics-700	Regular action	700	65,000	[40]
Breakfast Dataset	Kitchen action	10	1989	[8]
Charades	Indoors activities action	157	9848	[31]
AVA	Regular action	80	57,600	[35]
Epic-Kitchens	Kitchen action	149	432	[9]
Something-Something	Actions with objects	174	220,847	[34]
Moments in Time-Dataset	Regular action and event	339	1,000,000	[33]
Sport1-1M	Sport	487	1,200,000	[2]
ActivityNet	Regular action	200	20,000	[10]
EduNet	Classroom action	20	7851	-

**Table 5:** Positive and negative action categories of student and teacher.

	Positive Actions	Negative Actions
Student	Clapping	Arguing
	HandRaise	Eating_in_classroom
	Reading_Book	Gossip
	Sitting_on_Desk	Sleeping
	Standing	
	Talking	
	Writting_on_Textbook	
Teacher	Explaining_the_subject	Hitting
	Holding_Book	Slapping
	Sitting_on_Chair	Holding_Mobile_Phone
	Walking_in_Classroom	Holding_Sticks
	Writing_On_Board	

**Table 6:** Comparison of the two-stream I3D result on benchmark datasets and EduNet.

Model	Pre-Training	UCF101	HMDB51	EduNet
RGB-I3D	Kinetics-400	95.1%	74.3%	65.2%
Flow-I3D	Kinetics-400	96.5%	77.3%	68.8%
Two-stream I3D	Kinetics-400	97.8%	80.9%	72.3%

## Data Availability

The dataset will be available to download at a later date, in compliance with regulations online at https://github.com/vijetait/Classroom-Monitoring-Action-Dataset, accessed on 19 August 2021.
